# “DV Fatigue”: Work Stress and Officers’ Attitudes and Performance at Domestic and Family Violence Incidents

**DOI:** 10.1177/10778012241239944

**Published:** 2024-03-20

**Authors:** Emily Maple, Mark Kebbell

**Affiliations:** Griffith University, Brisbane, Queensland, Australia

**Keywords:** domestic and family violence, police, performance, attitudes, job stress

## Abstract

A self-report instrument was created to measure stress, attitudes, and performance of domestic and family violence (DFV) first responders in an Australian state. DFV-related stress negatively impacted officers’ attitudes and self-assessed performance. Higher DFV stress was predicted by the frequency and severity of DFV incidents, and the absence of lived experience. Negative attitudes were predicted by a shorter length of service and lower severity, and poorer performance by a longer length of service and lower perceived social support. Males reported higher stress and poorer performance than females. The findings reveal systemic issues that inhibit effective police response, emphasizing the need to address negative attitudes and “DV fatigue.”

Responding to domestic and family violence (DFV) poses significant challenges for law enforcement agencies (Johnson, 2004). DFV refers to a pattern of abusive behaviors that occur within spousal relationships, dating relationships, and family relationships (Hegarty et al., 2000). It encompasses various forms of abuse, including physical, sexual, emotional, psychological, and financial abuse (Hegarty et al., 2000). Police officers have faced criticism for their attitudes toward DFV and their involvement in a system that has at times failed to intervene (Goodman-Delahunty & Crehan, 2016; Meyer, 2011). DFV incidents represent a distinct and substantial stressor for frontline police officers (Maple & Kebbell, 2021). Stress reduction and improved attitudes to policing DFV, therefore, might be central to improving police responses to DFV. To achieve this, law enforcement agencies need a way to measure the level of stress experienced by officers responding to DFV incidents and to assess their attitudes toward policing such cases. In this article, we report on an attempt to conceptualize and analyze the relationships among stress, attitudes, and performance relating to DFV in a sample of frontline officers.

Our objective was to develop a survey tool that assesses frontline officers’ capacity to effectively respond to DFV. Strong capacity to respond to DFV is conceptualized in this regard includes (a) officers displaying positive and proactive attitudes toward police intervention, finding the role rewarding and in alignment with their policing philosophy; (b) possessing adequate mental and emotional resources, free from excessive stress; and (c) having the necessary practical resources and organizational support to maintain well-being, engagement, and effective performance. The survey tool offers a means to monitor progress over time and assess the effectiveness of intervention strategies.

## Contemporary Police Attitudes

Police agencies have been criticized for their attitudes toward DFV and the quality of their responses to incidents. Concerns include officers engaging in victim-blaming by questioning the credibility or actions of the survivor, being unwilling to intervene, and lacking proper knowledge and understanding of the dynamics of DFV (DeJong et al., 2008; Serrano-Montilla et al., 2023). Taken together, the literature suggests that generally, police have more negative attitudes to policing DFV; they find DFV incidents frustrating, and they dislike responding (e.g., Maple & Kebbell, 2021; Segrave et al., 2018). Importantly, attitudes appear to influence the quality of responses (Islam & Mazerolle, 2022; Zhao et al., 2018).

Examples of less effective police responses to DFV include officers discouraging victim-survivors from reporting incidents (e.g., due to an officer's caustic manner or lack of empathy), inadequate support offered to survivors, not responding to incidents in a timely fashion, and underestimating the severity of the situation or a failing to recognize coercive control (Goodman-Delahunty & Crehan, 2016; Meyer, 2011). Ineffective DFV responses further perpetuate a culture of silence and hinder the provision of necessary support and intervention. Additionally, police departments may have structural and organizational barriers that hinder effective responses to DFV, such as a lack of resources, limited training opportunities, or a lack of protocols or guidelines for handling DFV incidents.

However, there are some encouraging findings that have emerged in the past few years. Gill et al. (2019) reported that most participants (92.3%) in a sample of Canadian police officers considered DFV as a serious issue and supported a strong criminal justice response. A similar finding was reported by El Sayed et al. (2022) pointing toward a positive shift in attitudes among police officers. Nonetheless, it is evident that there is scope to enhance police responses and improve their attitudes. The solution to addressing problematic police attitudes and ineffective response to DFV remains unclear. While there are some useful but indirect methods, such as improving training for officers and investigating police misconduct, there is no clear-cut answer.

A limitation of current DFV and policing research is that it primarily focuses on training and sociodemographic predictors of police attitudes. While these are important factors, studies to date have neglected the broader context of occupational stress in relation to DFV incidents, which likely exerts a significant impact on officers’ attitudes (Maple & Kebbell, 2021). In our present research, we examine the relationship between police stress, DFV attitudes, and their influence on performance. We aim to gain a deeper understanding of the most impactful sources of stress relating to DFV, and how stress experienced by police officers can shape their attitudes and ultimately affect their job performance.

## Designing a Police Attitudes Scale

To gain a comprehensive understanding of and effectively address the issues surrounding police responses, it is important that we develop a conceptual framework and accurate measures for assessing police attitudes. There are existing measures available, such as those discussed by Gracia et al. (2018) and McPhedran et al. (2017), which focus on aspects of attitudes such as victim blaming and willingness to intervene. However, they do not consider officers’ emotional responses, their orientation toward investigation or service, their trust in the organization, and their perceptions of rewards and challenges in their role. These additional factors could be conceptualized as components of attitudes and may help us understand the impact on officers’ performance and to identify modifiable risk factors.

In developing a DFV Attitudes Scale, we drew upon qualitative data from interviews with general duties police officers and incorporated insights from the literature and existing measures. DFV attitudes are defined in the current study as cognitive and affective responses toward people, issues, or situations that are relevant to being a first responder to DFV incidents. This includes policing philosophy (investigation vs. service orientation), sources of satisfaction/dissatisfaction, beliefs about the importance of different types of DFV, appreciation for its complexity, endorsement of myths and stereotypes, and trust in the police organization.

The scale aimed to distinguish between positive attitudes (optimistic, invested, and prosocial) and negative attitudes (dissatisfied, disengaged, and cynical) regarding policing DFV. The assumption was that more positive attitudes would predict more positive behavior, and vice versa. It is important to note that these positive or negative attitudes are not labeled as “correct” or “incorrect” and do not reflect an officer's overall competence. Rather, they help identify a set of beliefs and values that were hypothesized to be associated with better police responses to DFV.

While the Attitudes scale design in the present study primarily takes a ground-up approach, incorporating relevant theories adds depth and validity to the measure. In this regard, several key theoretical constructs were integrated, including psychosocial safety climate, role strain, policing philosophy, and moral distress. These constructs provide a framework for understanding and assessing the instrument.

### Psychosocial Safety Climate

Psychosocial Safety Climate is a unifying framework of the dominant occupational stress models. Dollard and McTernan (2011) define Psychosocial Safety Climate as an organization's focus on managing employee psychological well-being and safety. It indicates how concerned management is about psychological health versus productivity, and how much mental health is prioritized. The advantage of the Psychosocial Safety Climate framework is that it considers broader influences (e.g., policies, leadership, the extent to which feedback and participation by employees is encouraged) to explain the development of work stress rather than placing undue responsibility on the individual (Dollard & McTernan, 2011). A poor psychosocial safety climate predicts more psychologically hazardous working conditions, which in turn influences the motivation and health of employees (Dollard & McTernan, 2011). Psychosocial safety climate is an important consideration for understanding police stress, attitudes, and the quality of frontline police responses to DFV.

### Policing Identity and Role Strain

Frontline police officers may experience role strain as they respond to a range of emergencies, each requiring different sets of skills. Role strain is a state of stress arising from multiple, complex, and possibly incongruent demands or obligations within one's job (Hardy & Conway, 1988). Huey and Ricciardelli (2015) found that most officers in a rural sample perceived their role within the service as either predominantly law enforcement or social workers. When officers’ role expectations conflicted with reality and they were required to perform tasks associated with a less desired role, the outcome appeared to be role strain characterized by feelings of frustration and demoralization. They observed negative effects on officer well-being and suggested that role strain may reduce the overall effectiveness of the police service.

Role strain may relate to policing identity or orientation. In the context of policing DFV for example, officers who are more service-oriented (i.e., derive satisfaction from being a supportive, counseling figure) tend to have more positive attitudes toward DFV and more empathetic and successful interactions with victims than those who are more investigation-oriented (i.e., place more importance on enforcing the law by arresting criminals; Balenovich et al., 2008; Barrett & Hamilton-Giachritsis, 2013; DeJong et al., 2008; Islam & Mazerolle, 2022). As such, the presence of role strain or the concept of policing identity is likely to play into their attitudes and perceptions of stress.

### Moral Distress

Moral distress is a condition of shame, guilt, and burnout resulting from witnessing, perpetrating, or failing to prevent harmful events (Papazoglou et al., 2020). The condition has been described in relation to soldiers, nurses, and police officers who are likely to be exposed to morally injurious situations (i.e., discharging a firearm in self-defense or triaging injured people in response to a disaster which may lead a person to feel conflicted about the ethicality of their actions). As noted by Papazoglou et al. (2020) workers in these professions are often functioning under “strained system conditions” which makes it difficult to act in accordance with one's values. Moral distress is associated with staff turnover and burnout (Meltzer & Huckabay, 2004). DFV incidents present many scenarios that could lead to moral distress for responding officers. A Magistrate's decision, for example, may defeat an officer's efforts to attain justice for an aggrieved, or they may feel uncertain about who is the perpetrator or victim. For officers who feel pressed beyond their capacity or are unable to meet the complex needs of an aggrieved or respondent, guilt and shame may also arise. This may then influence attitudes toward DFV and contribute to poorer outcomes.

## The Importance of Work Stress

General work stress in policing is associated with poor mental and physical health as well as impaired judgment and performance (Kop et al., 1999). Sources of police stress are typically categorized as operational (interacting with the public and attending potentially traumatic events) or organizational (administration, management, structure, and processes; Anshel, 2000). In the police stress literature, organizational stressors tend to be perceived as more stressful than operational factors (Anshel, 2000; Chan, 2001). Policing DFV involves aspects of both types of stress (Maple & Kebbell, 2021). Officers’ perceptions of stress, the development of attitudes, and perceptions of performance in relation to DFV incidents may depend on a combination of individual and environmental factors.

### Defining and Measuring Stress

Defining and measuring stress is crucial in understanding the impact of police job stress in relation to attitudes and responding to DFV incidents. Stress can be defined as a physiological and psychological response to external demands that exceed an individual's ability to cope. It is a complex and subjective experience. Measuring subjective perceptions of stress through a self-report questionnaire is one of several methods considered for the current study. While objective physiological measures like heart rate variability and skin conductance can provide valuable insights, it was important for the purpose of this study to identify specific sources of stress (i.e., operational vs. organizational) and provide a tool that is quick and simple to administer and score. Self-report data allows us to gain insights into officers’ personal experiences, enabling us to develop targeted interventions and support systems to mitigate the negative effects of stress and improve overall well-being. Future research in this domain could potentially gain valuable insights by incorporating objective physiological indicators to assess stress levels.

### Is DFV Uniquely Stressful?

Maple and Kebbell (2021) explored the perceptions of frontline police to investigate concerns of police becoming disengaged and desensitized to DFV. Officers reported that it was both the futility of attending DFV incidents and broader organizational factors that shaped their views. DFV incidents appeared to be disempowering for officers as they attended many minor incidents or attended the same addresses repeatedly with no positive outcome. DFV incidents were also frustrating for police officers due to the bureaucracy and heightened scrutiny associated with these incidents. The study suggested that responding to DFV represented a major and potentially unique source of daily stress and that some frontline police were not coping well. If left unaddressed, frustration itself may perpetuate the problem: frustration could lead to ill-health, low morale, and poor behavior (Johnson, 2004).

Although DFV is currently a major portion of what frontline police deal with, the likely contribution of DFV incidents to occupational stress and the link to impaired performance has received little attention. Existing measures of police stress such as the Operational Police Stress Questionnaire (PSQ-OP) and Organizational Police Stress Questionnaire (PSQ-ORG) by McCreary and Thompson (2006) do not capture DFV incidents specifically. The scale designed to measure DFV stress in the current study uses a similar format to the PSQ-OP and PSQ-ORG. Comparisons with an established measure allow us to determine whether DFV stress is indeed a separate construct and adds something unique to the pool of general police stress.

## Predictors of Attitudes, Stress, and Performance

The study also aimed to investigate other predictors of stress, attitudes, and performance in the context of policing DFV. It is well-established that general police stress has significant implications for both mental and physical health outcomes, as well as job performance. Individual factors, such as personality traits, preexisting mental health conditions, and social support, have also been found to play a role in influencing stress levels and related outcomes (Bianchi, 2018; Ozbay et al., 2007; Pole, 2008). Therefore, this study included these variables as control measures to account for their potential influence on the outcomes of interest. In addition to the fundamental control variables, our study focused on four crucial factors as potential indicators of stress, attitudes, and performance: officer gender, lived experience of DFV, and the frequency and physical severity of DFV incidents attended on the job.

### Officer Gender

There is debate about the influence of masculine police culture on officers’ attitudes toward DFV (Loftus, 2009). Female officers responding to DFV incidents display more patience, compassion, and proactive attitudes compared to male officers, indicating the potential impact of gender bias (Belknap, 1995; El Sayed et al., 2022; Robinson & Chandek, 2000; Serrano-Montilla et al., 2023). A cross-national survey by McPhedran et al. (2017) found that male officers in Australia were more likely to endorse police discretion and view DFV as a private matter, contrary to their female counterparts. Pereira Vieira et al. (2022) discovered that female military personnel in Portugal exhibited higher levels of disagreement with DFV stereotypes and were more inclined to intervene. However, McPhedran et al. (2017) and El Sayed et al. (2022) support the notion that male and female officers have more similarities than differences in their views on DFV. Additionally, both suggest that there has been a positive trend in recent years toward improved perceptions of DFV.

### Lived Experience

The influence of police officers’ personal experiences as victim-survivors of DFV on their perceptions of the issue as first responders remains unclear. While Serrano-Montilla et al. (2023) acknowledge the role of lived experience in shaping police attitudes, they do not specify its directionality. Other sectors, such as mental health, recognize and leverage the value of lived experience, as it fosters a shared understanding between service providers and users, reduces power imbalances, and improves service uptake and outcomes (Resnick & Rosenheck, 2008). Therefore, it is worth investigating whether lived experience correlates with more positive attitudes toward DFV and enhanced frontline officer performance when compared to those without such experiences.

### Frequency and Severity of Incidents

Higher perceived severity of DFV is associated with lower endorsement of myths (i.e., beliefs that minimize or justify DFV) and a greater willingness to intervene (Serrano-Montilla et al., 2023). Conversely, the presence of anchoring effects and working in an environment characterized by more frequent and higher levels of violence can potentially influence perceptions and desensitize officers to the risks involved (Kebbell, 2022). Is therefore reasonable to expect a dose effect whereby the frequency of attendance at DFV and the level of violence encountered have an influence on officer stress, attitudes, and performance—although the directionality is uncertain.

## Study Aims

In this study, we report on the development and preliminary evaluation of a novel self-report instrument designed to help police agencies gain an overview of frontline officers’ perceptions of stress and attitudes to DFV incidents and how these factors relate to self-assessed performance. Second, we wished to examine the effects of gender and lived experience. Third, we wished to examine predictors of DFV stress, attitudes, and performance through regression modeling. Based on the literature reviewed, the following hypotheses were posed: (a) Increased DFV stress would predict negative DFV attitudes; (b) Lower DFV stress and positive DFV attitudes would predict positive performance; (c) Higher frequency and more severe DFV incidents would predict increased DFV stress; (d) There would be a positive effect of being female and having lived experience on DFV stress, attitudes, and performance.

## Method

### Participants and Procedure

This research was completed as part of a project with the Queensland Police Service to investigate the possibility of frontline officers becoming disengaged and desensitized to DFV. A survey was sent to a 1,500 general duties officers in Queensland and was completed by 218 participants (54 women, 152 men) in October 2021. Data were collected on the instrument described here and a range of mental health outcomes as well as sociodemographic and personality factors. Following ethics approval, a stratified random sample of frontline officers from an Australian state was provided to the researchers by the police organization. An email was sent inviting officers to participate in an anonymous online survey about policing DFV and police well-being by accessing a URL. No incentives were offered for participation.

### Measures

Sociodemographic information was collected including gender, ethnicity, age, rank, education, marital status, years of service, frequency of attending DFV incidents (one per month or less to multiple times per shift), frequency of exposure to severely violent DFV incidents, general location (e.g., rural, urban), preexisting mental health conditions, and lived experience as a victim-survivor of DFV either in childhood or adulthood. A range of other variables were included in the survey (contact authors for more information). Those relevant to the research aims were general police stress (PSQ-OP, PSQ-ORG; McCreary and Thompson, 2006), a short measure of personality (10-Item Personality Inventory; Gosling et al., 2003), and social support (Interpersonal Support Evaluation List—shortened version; Cohen et al., 1985).

#### DFV Attitudes

A large pool of attitudinal statements was generated including reverse-scored items. The pool was reduced to 48 items based on relevance and readability. These are displayed in the Results section. Items such as “Many aggrieved make up stories or exaggerate to get their partner in trouble” and “I more often feel sorry for the respondent than the aggrieved” tap into the concept of officers experiencing moral distress. Similarly, items such as “A satisfying part of my job is listening and offering support” and “The most important part of my job is arresting criminals” are intended to reflect an officer's policing philosophy (i.e., service-oriented vs. investigation-oriented) which may also indicate the extent to which they experience role strain in responding to DFV. A number of items also tap into psychosocial safety climate including “Ticking the right boxes for DV is more important to senior management than staff well-being” and “I trust my organization to back me up with DV incidents.” Responses were measured on a 5-point scale (“*strongly disagree*” to “*strongly agree*”). Higher scores indicate more negative attitudes.

#### DFV Stress

Items for the DFV Stress Scale were generated around organizational stressors and operational stressors. Specific stressors were identified from the responses of officers gathered in the preliminary study (Maple & Kebbell, 2021) and wider literature. Participants were asked to indicate how stressful or frustrating they found each item over the past 6 months. Example operational stressors were: “Attending minor domestic disputes (e.g., raised voices) or false alarms,” and “Dealing with people who were intoxicated by drugs or alcohol.” Examples of organizational stressors are: “DV paperwork,” and “Your actions at a DV job being criticized.” Responses were measured on a 7-point scale (“*not at all*” to “*extremely*”) to allow for direct comparison with the PSQ-Org and PSQ-OP Scales. Higher scores indicate higher current levels of stress perceived in relation to DFV incidents.

#### DFV Performance

This scale was designed to capture the officers’ self-assessed use of interpersonal skills, compliance with procedure, and self-efficacy during DFV incidents. The items included positively and negatively worded questions about the frequency of behaviors. An example of positive items is “How often do you think you were you courteous and sensitive to the aggrieved?” An example negative item is “How often do you think you may have made procedural errors at a DV job?” Responses were measured on a 5-point scale (“*never*” to “*every time*”). Negatively worded items were reverse scored so that higher overall scores reflected better self-assessed performance at DFV incidents.

### Data Analysis

IBM^®^ SPSS^®^ was used for all statistical analyses. Principal components analyses were used for item reduction and factor extraction. Cronbach's alpha was used to provide a measure of reliability for each scale. Univariate statistics were calculated separately for items. T-tests were conducted to determine whether responses varied by gender or lived experience. Analyses of relationships between continuous and categorical variables relating to the hypotheses were conducted using Pearson's product–moment correlations, point-biserial correlations, and hierarchical multiple regression. Regression assumptions were examined prior to interpreting results. In cases where values were missing for one or both of a pair of variables, the cases were excluded from analyses.

## Results

### Sample Characteristics

There were 218 general duties police officers included in the sample (see Table 1). Most respondents were male (69.7%), ranked senior constable, aged 35–54 (77%), based in urban (32%) or suburban areas (35%), and attended one or more DFV incidents per shift (56.4%). One-quarter of officers stationed in rural and remote areas often or always attended DFV with extreme physical violence or severe injuries compared to 15.5% of officers from outer suburban regions, 11.5% of officers from country towns, and 8.8% of officers from inner city areas. Around one-fifth (21.2%) had lived experience as a victim of DFV in their lifetime.

**Table 1.** Sociodemographic Characteristics of Participants.

Sample characteristics	Total sample (*n = *218)
	*n* (%)
Gender	
Female	54 (24.8)
Male	152 (69.7)
Marital status	
Single	19 (8.7)
Married/partnered	178 (81.7)
Divorced/widowed	17 (7.8)
Other	4 (1.8)
Age	
18–24	3 (1.4)
25–34	36 (16.5)
35–44	96 (44.0)
45–54	72 (33.0)
55–64	11 (5.0)
Education	
High school Yr 10	8 (3.7)
High school Yr 12	48 (22.0)
TAFE	79 (36.2)
University or postgrad	71 (32.6)
Rank	
Constable	42 (19.3)
Senior constable	124 (56.9)
Sergeant	34 (15.6)
Senior sergeant	15 (6.9)
Frequency of DFV jobs	
≤ One per month	20 (9.2)
Several per month	21 (9.6)
≥ One per week	53 (24.3)
≥ One per shift	123 (56.4)
Frequency of severely violent DFV jobs	
Never	10 (4.6)
Rarely	71 (32.7)
Sometimes	107 (49.3)
Often	19 (8.8)
Always	10 (4.6)
Lived experience as a victim of DFV	
Yes	46 (21.2)
No	160 (73.7)
Prefer not to say	11 (5.1)

*Note.* DFV = domestic and family violence.

### DFV Attitudes Scale

A principal components analysis (PCA) was run on the 48 items concerning attitudes toward DFV. Examination of the eigenvalues and scree plots with a Varimax orthogonal rotation indicated a six-component structure. However, the components were not easily interpretable. A forced-factor extraction was then performed to extract four components, and this improved the interpretability of the components. There were strong loadings of beliefs about DFV importance in Component 1, cynicism toward the aggrieved in Component 2, policing identity in Component 3, and organizational trust items in Component 4. The final DFV Attitudes Scale consisted of 24 items with Cronbach's Alpha, α = .87, indicating good internal consistency. See Table 2 for the final scale including component loadings and communalities.

**Table 2.** DFV Attitudes Rotated Structure Matrix for PCA With Varimax Rotation of a Six-Factor Scale.

Items	1. DFV importance	2. DFV cynicism	3. Policing identity	4. Organizational trust	Communalities	Item *M* (*SD*)	Component *M* (*SD*)
Physical DV is more important for police to deal with than controlling behaviors.	**0**.**76**	0.03	0.04	−0.07	.58	2.56 (1.02)	*DV importance* 2.71 (0.66)
Verbal violence is not that serious.	**0**.**75**	0.16	0.09	−0.10	.61	2.28 (0.78)	
Most DV incidents are petty and minor, we could be spending time on more important things.	**0**.**64**	0.39	0.33	0.09	.68	2.88 (1.10)	
I prefer to jump straight to the important facts rather than listening to people complain	**0**.**62**	0.08	0.43	0.06	.58	2.96 (1.05)	
Most DV is just a one-off fight that got out of hand.	**0**.**56**	0.40	−0.04	0.05	.47	2.31 (0.85)	
I feel less motivated to help when the aggrieved is uncooperative.	**0**.**55**	0.20	0.14	0.34	.46	3.44 (1.06)	
I feel less motivated to help when the aggrieved is intoxicated.	**0**.**53**	0.25	0.23	0.24	.48	2.83 (1.07)	
Most DV would be better dealt with by another unit or agency.	**0**.**50**	0.23	0.30	0.23	.45	3.50 (1.10)	
DV is a family's private business, police should not meddle in it.	**0**.**47**	0.37	0.00	−0.06	.36	1.63 (0.73)	
Women tend to take advantage of DV.	0.15	**0**.**78**	0.29	−0.07	.72	3.06 (1.02)	*DV cynicism* 2.78 (0.64)
Many aggrieved make up stories or exaggerate to get their partner in trouble.	0.10	**0**.**69**	0.28	−0.10	.58	3.33 (0.91)	
I more often feel sorry for the respondent than the aggrieved.	0.24	**0**.**64**	−0.06	0.05	.47	2.24 (0.76)	
Men are just as likely as women to be victims of DV.	−0.01	**0**.**64**	−0.26	0.10	.48	3.54 (1.08)	
I can understand why some respondents lose control, the aggrieved pushed them too far.	0.24	**0**.**61**	−0.03	0.09	.44	2.61 (1.10)	
Most aggrieved are genuine. (R)	0.23	**0**.**57**	0.35	0.10	.51	2.75 (0.93)	
Some aggrieved seem to like the abuse because they refuse to leave.	0.25	**0**.**53**	−0.04	0.12	.36	1.88 (0.87)	
A satisfying part of my job is simply listening and offering support. (R)	−0.02	0.10	**0**.**80**	0.17	.68	3.09 (1.04)	*Policing identity* 3.14 (0.75)
I feel satisfied that I helped the aggrieved or respondent even if there was no real outcome. (R)	0.07	−0.08	**0**.**69**	0.21	.54	2.91 (0.93)	
The most important part of my job is arresting criminals.	0.28	0.09	**0**.**56**	0.10	.41	2.88 (1.08)	
Police officers should not have to be social workers.	0.24	0.00	**0**.**56**	0.24	.43	3.66 (1.01)	
Ticking the right boxes for DV is more important to senior management than staff well-being.	−0.03	0.16	0.09	**0**.**82**	.70	4.19 (0.97)	*Organizational trust* 3.95 (0.72)
I trust my organization to back me up with DV incidents. (R)	0.09	−0.09	0.18	**0**.**78**	.66	3.96 (1.01)	
I feel that I need to cover my back when it comes to DV incidents.	−0.06	0.24	0.15	**0**.**64**	.49	4.50 (0.69)	
My judgement is respected by senior management at DV incidents. (R)	0.18	−0.14	0.25	**0**.**60**	.48	3.14 (1.10)	

*Note.* DFV = domestic and family violence; PCA = principal components analysis.

(R) denotes reverse-scored items. Major loadings for each item are bolded. Responses scored from 1 to 5.

Responses indicated that responses on organizational trust and policing identity were most negative, while responses on cynicism toward the aggrieved and beliefs about the importance of DFV were more neutral. In terms of organizational trust, a high proportion of participants agreed that ticking the right boxes for DFV was more important to senior management than staff well-being (79.5%) and felt they needed to cover their backs when it comes to DFV (92.5%). Sixty percent of participants agreed that police officers should not have to be social workers while 32.9% felt that listening and offering support was a satisfying part of their role. Few endorsed the belief that DFV is a family's private business (1.2%), while 82% agreed that controlling behaviors were of equal importance to physical violence for police to deal with, and 92.6% agreed that verbal violence was serious. However, 46.9% agreed that many aggrieved make up stories or exaggerate to get their partner in trouble. Thirty percent agreed they felt less motivated to help when the aggrieved was intoxicated.

### DFV Stress Scale

A PCA was run on the 28-item DFV Stress Scale that measured perceptions of DFV incident stress. Again, a forced-factor extraction was used with two components to be retained in line with the theoretical divide between organizational and operational stressors. The PCA revealed that a two-component solution was unsatisfactory as some items were not loading onto either factor. Therefore, the PCA was rerun with a three-component forced-factor extraction. The three-component solution met the interpretability criterion and was retained. The interpretation of the data showed strong loadings of organizational items on Component 1, routine operational items on Component 2, and critical incidents on Component 3. Component loadings and communalities of the rotated solution are presented in Table 3. Internal consistency of the final 26-item scale was considered good based on Cronbach's alpha (*α =* .87).

**Table 3.** DFV Stress Rotated Structure Matrix for PCA With Oblimin Rotation of a Three-Factor Scale.

	1. Organizational factors	2. Critical incidents	3. Routine operational factors	Communalities	*M* (*SD)*	*M* (*SD)*
DV bureaucracy or “red tape.”	**0**.**91**	−0.03	0.03	.76	5.42 (1.80)	*Organizational* 4.84 (1.59)
Feeling like your actions are being scrutinized when you attend DV incidents.	**0**.**89**	−0.05	0.00	.76	5.41 (1.94)	
Excessive pressure and expectations at a DV job.	**0**.**84**	−0.04	−0.12	.82	5.30 (1.83)	
Your actions at a DV job being criticized.	**0**.**82**	0.04	−0.04	.75	5.22 (2.0)	
DV paperwork.	**0**.**82**	−0.02	−0.05	.71	5.47 (1.89)	
Disagreeing with DV policies and procedures.	**0**.**74**	0.02	−0.11	.69	4.67 (2.03)	
Fearing that you might lose your job because of something you did or did not do at a DV job.	**0**.**66**	0.05	−0.12	.58	4.63 (2.22)	
Inadequate support from supervisors in relation to DV incidents.	**0**.**59**	0.29	0.08	.47	3.44 (1.94)	
Not having enough equipment or resources to do your job at DV properly (e.g., cars, facilities, support services for victims).	**0**.**53**	0.19	−0.17	.56	4.20 (2.09)	
Not enough staff to cope with the amount of DV calls in your area.	**0**.**49**	0.29	−0.09	.51	4.64 (2.11)	
Seeing fatalities (i.e., suicide, homicide) in a DV situation.	0.06	**0**.**90**	0.14	.76	4.02 (2.06)	*Critical Incident* 4.04 (1.56)
Seeing sexual violence in a DV situation.	0.06	**0**.**89**	0.08	.77	3.91 (1.96)	
Dealing with people in distress at DV incidents (i.e., severe injuries, traumatized people).	0.09	**0**.**77**	−0.11	.74	3.83 (1.77)	
Being threatened or assaulted at a DV job.	0.13	**0**.**63**	−0.20	.65	3.94 (2.01)	
Using force against an aggrieved or respondent in self-defense.	0.17	**0**.**62**	−0.19	.66	3.59 (1.99)	
Seeing children affected by DV.	−0.12	**0**.**46**	−0.44	.46	4.89 (1.64)	
Aggrieved who will not help themselves.	0.10	−0.15	**−0**.**85**	.76	4.92 (1.84)	*Routine operational* 4.36 (1.56)
Dealing with DV among people from low-income areas (i.e., unemployed, uneducated).	−0.12	0.17	**−0**.**80**	.66	3.53 (2.10)	
Aggrieved or respondent not cooperating with police.	0.10	−0.04	**−0**.**79**	.72	4.82 (1.83)	
Dealing with DV among people who are intoxicated by drugs or alcohol.	−0.01	0.22	**−0**.**75**	.74	4.62 (1.92)	
Playing the role of a counselor or social worker at DV incidents.	0.14	0.04	**−0**.**73**	.72	4.69 (2.08)	
Aggrieved taking advantage of the justice system.	0.19	−0.16	**−0**.**71**	.64	4.75 (1.98)	
Attending minor domestic disputes (e.g., raised voices) or false alarms.	0.23	−0.21	**−0**.**67**	.60	3.90 (2.02)	
Feeling like your time and effort has been wasted at DV incidents.	0.26	−0.06	**−0**.**66**	.70	4.74 (1.89)	
Dealing with people from a different culture at DV incidents.	−0.10	0.28	**−0**.**63**	.52	3.04 (1.95)	
Attending repeated calls for the same aggrieved and respondent.	0.26	0.05	**−0**.**58**	.64	4.64 (1.85)	

*Note.* DFV = domestic and family violence; PCA = principal components analysis.

Major loadings for each item are bolded. Responses scored from 1 to 7.

The most stressful DFV items that officers identified were paperwork, bureaucracy, or “red tape,” feeling like actions are being scrutinized, excessive pressure and expectations, actions being criticized, aggrieved who will not help themselves, seeing children affected by DFV, aggrieved or respondent not cooperating with police, aggrieved taking advantage of the justice system, and feeling like time and effort was wasted. In general, organizational DFV stressors elicited higher perceived stress than routine operational or critical incident stressors.

### DFV Performance Scale

A final PCA was run on the 14-item DFV Performance Scale. PCA revealed three components that had eigenvalues greater than one. A Varimax orthogonal rotation was employed to aid interpretability. The interpretation of the components was consistent with the performance characteristics the scale was designed to measure. Component loadings and communalities of the rotated solution are presented in Table 4. The final DFV Performance Scale consisted of 14 items with Cronbach's Alpha, α = .87, indicating good internal consistency.

**Table 4.** DFV Performance Rotated Structure Matrix for PCA With Varimax Rotation of a Three-Factor Scale.

Items	Principal components					
	1. Negative performance	2. Interpersonal skills	3. Decision-making self-efficacy	Communalities	*M (SD)*	*M (SD)*
How often do think you may have been too aggressive at a DV job? (R)	**0**.**79**	0.18	0.04	0.66	1.85 (0.76)	*Negative performance* 3.83 (0.57)
How often do you think you came down too hard on the aggrieved? (R)	**0**.**76**	0.22	0.18	0.67	2.04 (0.74)	
How often do you think you came down too hard on the respondent? (R)	**0**.**75**	0.19	0.08	0.61	2.23 (0.79)	
How often do you think you may have been too impatient at a DV job? (R)	**0**.**75**	0.29	0.00	0.64	2.37 (0.84)	
How often do you think you may have been unsympathetic at a DV job? (R)	**0**.**69**	0.34	−0.05	0.59	2.43 (0.92)	
How often do you think you may have handled a DV job poorly overall? (R)	**0**.**64**	−0.05	0.41	0.39	1.89 (0.66)	
How often were you not satisfied with the decisions you made at a DV job? (R)	**0**.**55**	−0.13	0.26	0.58	2.34 (0.96)	
How often do you think you may have made procedural errors at a DV job? (R)	**0**.**54**	−0.23	0.53	0.62	2.25 (0.73)	
How often do you think you were you courteous and sensitive to the respondent?	0.21	**0**.**86**	0.17	0.81	4.07 (0.64)	*Interpersonal skills* 4.14 (0.56)
How often do you think you were you courteous and sensitive to the aggrieved?	0.17	**0**.**81**	0.27	0.76	4.31 (0.65)	
How often do you think you were tolerant with people at a DV job even if they were difficult?	0.14	**0**.**75**	0.27	0.66	4.05 (0.60)	
How often do you think you followed all DV procedures correctly?	0.11	0.25	**0**.**76**	0.65	4.37 (0.66)	*Decision-making self-efficacy* 4.2 (0.50)
How often do you think you handled a DV job well overall?	0.01	0.30	**0**.**76**	0.66	4.11 (0.54)	
How often were you satisfied with the decisions you made at a DV job?	0.16	0.31	**0**.**67**	0.58	4.24 (0.64)	

*Note*. DFV = domestic and family violence; PCA = principal components analysis.

(R) denotes reverse-scored items. Major loadings for each item are bolded. Responses scored from 1 to 5.

Most officers reported positive perceptions of their performance at DFV incidents. They reported being courteous and sensitive toward the aggrieved (96.9%), feeling satisfied with their decisions (94.4%), and following all DFV policies and procedures (96.3%) most of the time or every time they attended a job. Most officers reported undesirable behaviors such as aggression, impatience, lack of sympathy, and procedural errors as never or almost never occurring. However, a notable proportion of officers reported occasionally (or more frequently) being too hard on the respondent (33.5%), being too impatient (45.4%), making procedural errors (33.5%), and feeling unsatisfied with their decisions (34.2%).

### The Effect of Gender and Lived Experience

Bivariate statistics were used to calculate differences by gender and lived experience. There was no significant effect of gender or lived experience on DFV attitudes scores, *t*(154) = −1.82, *p* = .072; *t*(151) = 1.38, *p = *.09. There was a significant effect of gender on DFV Stress scores, *t*(155) = −2.38, *p* = .032, with male officers (*M = *93.4*, SD = *34.5) reporting higher stress than female officers (*M = *77.6*, SD = *40.2)*.* There was also a significant effect of lived experience on DFV stress scores, *t*(152) = 1.73, *p* = .046, with individuals who have lived experience of DFV (*M = *77.67, *SD *= 44.03) reporting lower DFV stress than individuals who did not have lived experience (*M = *91.92, *SD *= 33.50). There was a significant effect of gender on DFV performance, *t*(153) = 2.26, *p* = .025, with female officers (*M = *4.12*, SD = *0*.*34*)* reporting slightly more frequent good performance than male officers (*M = *3.93*, SD = *0.47). There was no significant effect of lived experience on DFV performance, *t*(150) = −1.8, *p = *.071.

## Hierarchical Regression Analyses

A series of hierarchical regressions were run with the Attitudes, Stress, and Performance Scales described above entered as dependent variables. Gender was included in each regression model as a covariate, otherwise, only variables (including controls) that significantly correlated with dependent variables were entered. Descriptive statistics and correlations are presented in Table 5. Assumptions were checked prior to interpreting the analyses.

**Table 5.** Descriptive Statistics and Correlations for Study Variables.

Variable	*n*	*M*	*SD*	1	2	3	4	5	6	7	8	9	10	11	12	13	14	15	16	17
1. General police stress	194	154.08	49.64	–																
2. Male gender	206	0.74	0.44	.19**	–															
3. Rural location	218	0.33	0.47	0.08	0.05	–														
4. University education	218	0.33	0.47	−.17*	−0.13	0.07	–													
5. Length of service	215	13.94	9.39	0.07	.17*	0.08	0.04	–												
6. Preexisting MH condition	199	0.19	0.39	.24**	0.00	0.03	−0.03	.181*	–											
7. Extraversion	140	4.30	1.44	−0.06	−.20*	−0.08	−0.05	−.205*	−0.03	–										
8. Emotional stability	140	5.41	1.20	−.27**	−0.06	−.22**	0.11	−0.14	−.38**	0.12	–									
9. Conscientiousness	140	5.83	0.98	−.17*	0.02	−0.06	0.05	0.07	0.02	−0.03	.24**	–								
10. Openness	140	4.73	1.14	−0.03	−0.01	−0.07	−0.09	−.20*	0.13	.29**	0.06	.22**	–							
11. Agreeableness	140	3.81	0.85	−0.07	−0.03	0.16	0.07	0.10	0.05	−0.10	0.01	0.10	−0.12	–						
12. Social support	139	62.57	14.20	−.28**	−0.12	−0.14	−0.01	−0.17	−.27**	.21*	.20*	0.13	−0.03	−0.09	–					
13. Lived experience of DFV	206	0.22	0.42	−0.06	−.14*	−0.04	−0.08	−0.03	/0.05	0.05	0.01	0.04	0.08	−0.02	−0.10	–				
14. Frequency of DFV jobs	217	3.29	0.98	0.06	−0.01	−.24**	−0.10	−.37**	−0.07	0.08	.29**	0.15	.204*	−0.13	−0.04	0.05	–			
15. Severity of DFV jobs	217	2.76	0.85	.15*	0.10	−0.05	0.00	0.05	−0.04	0.05	−0.01	0.07	.25**	−0.12	−0.11	−0.04	.30**	–		
16. DFV attitudes	162	3.00	0.50	.36**	0.14	−0.08	−0.11	−.23**	−0.02	−0.16	−0.06	0.03	−0.01	0.04	0.11	−0.11	0.06	−0.15	–	
17. DFV stress	163	115.77	36.38	.78**	.19*	0.08	−0.15	−0.03	0.08	−0.03	−.17*	−0.07	0.05	−0.08	−0.07	−.16*	.20*	.28**	.46**	–
18. DFV performance	161	3.98	0.45	−.18*	−.18*	−.18*	0.10	−.20*	−0.05	0.02	.34**	0.16	0.14	−0.01	.20*	0.15	.25**	0.09	−.28**	−.19*

*Note.* DFV = domestic and family violence.

*p < .05. **p < .01.

### The Prediction of DFV Stress

A hierarchical multiple regression was run to determine the prediction of DFV stress from significant correlates. See Table 6 for full details on each regression model. The full model was significant; *F*(7, 120) = 45.178, *p* < .001, *R^2^* *= *.725, adj.*R^2^* = .709. Block I with the inclusion of control variables (general police stress, gender, emotional stability) was significant; *F*(3, 124) = 67.820, *p* < .001, *R^2^* *= *.621, adj.*R^2^* = .612, and accounted for a large proportion (61.2%) of the variance in DFV stress. Introducing lived experience of DFV, frequency of DFV incidents, and severity of DFV incidents in Block II explained an additional 5.9% of the variance and the change in *R^2^* was significant; *F*(3, 121) = 7.462, *p* < .001, *R^2^* *= *.680, adj.*R^2^* = .665. Adding DFV attitudes to the regression model in Block III explained an additional 4.4% of the variance in *R^2^* and this change was significant, *F*(1, 120) = 19.403, *p* < .001. When interpreting the full model, the strongest predictor was General Police Stress which showed a large effect size based on semipartial correlations (.71). Higher general police stress, higher frequency of attending DFV incidents, greater severity of DFV incidents, and more negative attitudes predicted higher perceived stress from DFV. Having lived experience with DFV predicted lower perceived stress from DFV.

### The Prediction of DFV Attitudes

The full model with DFV attitudes as the dependent variable was significant; *F*(5, 150) = 15.424, *p* < .001, *R^2^* = .340, adj.*R^2^* = .318. Block I with the inclusion of control variables (general police stress, gender, length of service) was significant; *F*(3, 152) = 12.053, *p *< .001, and accounted for 19.2% of the variance in DFV attitudes. Introducing the severity of DFV incidents in Block II accounted for an additional 5.2% of the variance in DFV attitudes and the change in *R^2^* was significant, *F*(1, 151) = 10.289, *p* < .002. Introducing DFV stress in Block III accounted for an additional 9.6% of the variance and the change in *R^2^* was significant, *F*(1, 150) = 21.771, *p* < .001. As shown in Table 5, the model demonstrated that higher DFV stress predicted more negative DFV attitudes. Longer service and greater frequency of severely violent DFV incidents predicted more positive DFV attitudes.

### The Prediction of DFV Performance

**Figure 1.**
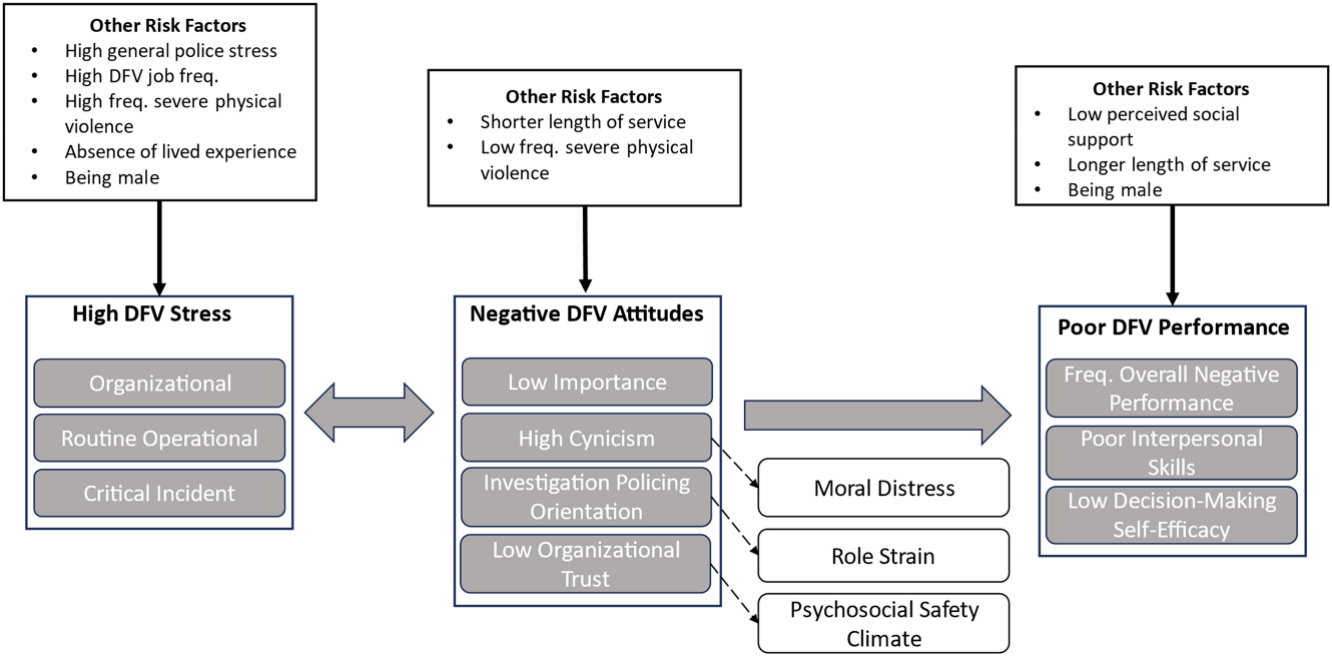
Mapping predictors and relationships among DFV stress, attitudes, and performance.

*Note.* DFV = domestic and family violence.

As shown in Table 5, the full model with DFV performance as the dependent variable was significant, *F*(7, 125) = 4.331, *p *< .001, and explained 29% of the variance in DFV performance. Block I including only control variables was significant; *F*(7, 125) = 4.331, *p *< .001, accounting for 19.5% of variance in DFV performance. The change in *R^2^* was not significant when DFV stress was introduced, *F*(1, 124) = 3.315, *p = *.071. When DFV attitudes was entered in Block III, the change in *R^2^* was significant, *F*(1, 123) = 12.702, *p *≤ .001 and this accounted for an additional 7.4% of the variance in DFV performance. Examination of the coefficients table for the full model showed that length of service, social support, and DFV attitudes were significant predictors of DFV performance. Longer service (*d = *.19), more positive perceptions of social support (*d* = .15), and more positive DFV attitudes (*d = *.27) predicted better self-assessed performance. Figure 1 illustrates the interconnectedness of various concepts, factors, and variables examined in this study, providing a visual representation of their suggested relationships.

## Discussion

A self-report instrument was developed to explore and quantify police officers’ experiences with DFV incidents. The study conceptualized and analyzed stress, attitudes, and performance in frontline officers, offering insights to improve their effectiveness and well-being. This instrument has strong psychometric properties and identifies a profile that predicts self-assessed job performance in DFV cases. By identifying key factors, this study provides a roadmap for police agencies to enhance their response to DFV and support officers in the field.

### DFV Stress in Frontline Police and Recommendations for Its Management

The DFV Stress Scale demonstrated a three-component structure with items loading onto organizational factors, operational factors, and critical incidents. This structure aligns with the theoretical divide between operational and organizational demands in policing (Anshel, 2000). Perceived stress was highest for organizational factors which is consistent with the literature (Anshel, 2000). However, these results add nuance to our understanding of police operational stress with regards to responding to DFV as it appears to naturally cluster into either acute (critical incidents or potentially traumatic events) or routine (common, everyday annoyances that are par for the course when interacting with the public). The implication is that both forms need to be managed.

The hypothesis that higher DFV stress would predict more negative attitudes was supported. As regression modeling cannot establish causality, the relationship between stress and attitudes could be bidirectional. Officers reported stress associated with DFV to be higher than other police work-related stressors. It is demonstrated that a combination of routine operational, serious critical incident, and organizational stressors influence, officers’ negative attitudes in relation to DFV.

Higher DFV stress among police officers may predict more negative DFV attitudes due to several factors. First, repeated exposure to DFV incidents can lead to increased frustration, apathy, and cynicism which may desensitize officers to the severity of DFV and result in a more negative perception of DFV cases (Maple & Kebbell, 2021). Second, excessive stress can lead to exhaustion and detachment (indicators of burnout), which may contribute to negative attitudes. Additionally, the stress associated with dealing with DFV situations may exacerbate negative emotions and biases, leading to more negative attitudes toward DV.

For the police organization, the “big ticket” items that can be modified to reduce routine organizational stress among frontline staff appear to be unnecessary paperwork and bureaucracy. Scrutiny, pressure, and criticism should be managed in such a way that it is fair and positive work is acknowledged. Operational stressors—whether acute or routine—are inherent to the job and are more challenging to address. Options may include strengthening support systems within the organization, stress management interventions, and enhancing new recruits’ psychological preparedness for DFV incidents. It is important that DFV stress among frontline police is addressed as a priority as officers appear unable to function effectively under current working conditions.

Against expectations, DFV stress did not directly predict performance. However, DFV stress appears to be the main predictor of attitudes which in turn predicts performance. The relationship between better attitudes and better performance could be explained by the role of empathy and interpersonal skills. Police officers with more positive attitudes toward DFV may possess higher levels of empathy, which would enable them to establish rapport and effectively engage with DFV victim-survivors. This, in turn, can positively impact their performance in providing support and assistance to victim-survivors. Additionally, officers with better attitudes may also be more motivated to continuously improve their skills and knowledge, leading to better overall performance.

### Shaping Positive Attitudes Among Officers

Officers’ attitudes toward DFV comprised four components: cynicism, importance, police identity, and organizational trust. As hypothesized, more negative DFV attitudes predicted poorer self-assessed performance. This finding suggests that attitudes are a key pathway to improving frontline police responses to DFV and we discuss this later.

An encouraging finding was that most officers agreed that DFV was important and serious (including verbal violence) and that DFV was a police matter with only 1.2% of the sample expressing a contrary opinion. This differs from earlier studies in which beliefs about DFV being a private matter were relatively prominent among police officers (DeJong et al., 2008; Loftus, 2009; Sinden & Stephens, 1999). It also contrasts with McPhedran et al.'s survey findings (2017) in which 23.2% of officers agreed that DFV was a family matter. Overall, the study findings support El Sayed et al.'s (2022) conclusion that police attitudes have shifted away from outdated beliefs and toward advocating for police intervention.

Items loading onto cynicism referred to beliefs and feelings toward the aggrieved with higher (i.e., more negative) scores indicating greater distrust of the aggrieved. Survey responses were somewhat supportive of cynical attitudes toward DFV with almost half the sample agreeing that many aggrieved make up stories or exaggerate to get their partner in trouble. This is consistent with attitudes reported in the literature (e.g., Maple & Kebbell, 2021; Myhill & Johnson, 2016; Segrave et al., 2018; Stewart et al., 2013). One explanation is that police are misinformed about the dynamics of DFV and how chronic abuse and coercive control can lead to false allegations from either party (Myhill & Johnson, 2016; Stark, 2007). Having a cynical attitude might influence officers’ behavior toward aggrieved and make them reluctant to arrest perpetrators who violate a protection order. Alternatively, it is possible that police officers see genuine issues that research is not capturing or reporting. Importantly, many officers feel they are unable to act in accordance with their beliefs and values, which leaves them at risk of moral distress (Papazoglou et al., 2020). As such, further clarification should be sought around police perceptions of false allegations in relation to DFV.

In terms of police identity, more officers than not endorsed an investigation-oriented view of their role which is consistent with Balenovich et al. (2008) and DeJong et al. (2008). A subgroup of officers appeared to find the service-oriented aspects of their role rewarding. Having to fill multiple roles at once (e.g., counselor and investigator) or being forced to complete tasks that align with a nonpreferred role may lead to diminished effectiveness and role strain. This is a difficult issue to address. One option is to recruit and train service-oriented police or to delegate DFV crisis response to those officers who are service-oriented. As noted above, enhancing psychological preparedness among recruits may also mitigate the issue to some extent. Another option is to involve other providers with specialist competencies (e.g., mental health nurses and social workers) in the crisis response. This would contain the role of frontline officers more strictly to peacekeeping, investigation, and law enforcement instead of asking them to wear many hats, some of which the majority are possibly ill-suited to. There have been trials of coresponder models applied to mental health crisis response, alleviating the pressure on police. A coresponder model may provide multiple benefits, such as reducing role strain and overall stress for officers while better meeting the complex needs of those who perpetrate or are victimized by DFV and their families.

The final component of DFV attitudes was organizational trust. Officers expressed a high degree of distrust toward the police organization. Officers felt they needed to “cover their backs” in relation to DFV jobs and that ticking the right boxes was more important to senior management than staff well-being. This indicates a need for an improved psychosocial safety climate within the police service.

### Frequency and Severity of DFV Incidents

The hypothesis that higher frequency and severity of DFV incidents would predict higher stress was supported. One implication of this finding is that frontline officers in regions with a higher concentration of DFV incidents or where they are more often exposed to severe physical violence (e.g., rural and remote communities) are more vulnerable to experiencing high levels of stress.

An unexpected finding was that higher severity of DFV incidents predicted more positive attitudes. One explanation for this could be that exposure to more severely violent DFV incidents heightens officers’ awareness and appreciation for the importance of DFV as a police matter. Physical violence with evidence of trauma is more straightforward for the criminal justice system to investigate and prosecute. Furthermore, repeated exposure to low-level violence may increase frontline officers’ complacency and sense of futility in their role (Stark, 2012). An alternative interpretation may be that officers with more negative attitudes perceive DFV incidents as less severe which would align with the anchoring effects seen in Kebbell (2022).

### Gender and Lived Experience

We hypothesized that there would be a positive effect of gender and lived experience on DFV stress, attitudes, and performance. Against expectations and in contrast to McPhedran et al. (2017) and El Sayed et al. (2022), officers’ overall attitudes toward DFV did not significantly differ by gender or lived experience. The lack of gender differences in attitudes is surprising, considering previous research has demonstrated that male police officers have less supportive attitudes toward DFV compared to their female counterparts. However, it is important to note that attitudes are complex and can be influenced by various factors such as cultural/social norms and individual predispositions. Some possible explanations are differences in sample size or composition and differences in instruments used to measure attitudes. Another possibility is that societal and cultural factors have changed over time, reducing the effects of gender bias among police officers.

The study found that perceptions of performance slightly but significantly differed by gender, with male officers reporting lower self-assessed performance than female officers. This indicates a potential gender disparity in how officers perceive their own performance in dealing with DFV cases. Furthermore, female officers and those with lived experience of DFV reported significantly lower perceptions of stress. One possible explanation could be that female officers may possess greater empathy and interpersonal skills, which could enhance their ability to effectively respond to and handle sensitive situations involving DFV (Sun, 2007).

Furthermore, research has shown that female officers may be more inclined to adopt community-oriented (i.e., service-oriented) policing strategies, which prioritize building relationships and trust with the community. This approach can positively impact their interactions with victims of DFV and contribute to lower stress levels and higher perceptions of performance (Stalans & Finn, 2000). Additionally, it is important to consider the influence of societal and cultural norms. Female officers may face societal expectations and pressures to prove their competence in traditionally male-dominated fields, leading them to exert more effort and diligence in their work, which could result in higher perceptions of performance (Sun, 2007). However, it is crucial to note that these are potential explanations and further research is needed to fully understand the underlying mechanisms and factors influencing the differing perceptions of performance between male and female officers in DFV incidents.

Lived experience as police officers may predict lower stress levels for several reasons. Firstly, officers with lived experience may have a deeper understanding and empathy toward victims, making them more effective at connecting and communicating with survivors (Resnick & Rosenheck, 2008). This ability to relate to victims may help reduce stress levels by fostering a sense of purpose and fulfillment in their work. This heightened awareness can contribute to a greater sense of control and confidence, which in turn may reduce stress levels. However, the impact of lived experience on attitudes or performance may vary. While it can enhance empathy and understanding, it may not necessarily translate into significant differences in attitudes or performance.

An incidental finding was that a longer length of service predicted more positive attitudes but poorer self-assessed performance. One possible explanation could be the effect of burnout or compassion fatigue. As police officers accumulate more years of service, they may become more exposed to the traumatic and challenging nature of DFV incidents, leading to greater understanding and appreciation for DFV but potentially greater emotional exhaustion and reduced job satisfaction (Stanetić & Tesanović, 2013). The presence of burnout could affect their overall performance in handling DFV cases, despite having more positive attitudes.

### Limitations and Future Directions

Although our study produced some novel insights, limitations must be acknowledged. The sample size was relatively small with a low response rate and therefore the results may not generalize to the larger population of frontline police. However, the sample is representative of the current gender and geographical distribution officers in the state. Another limitation is that we did not control for social desirability bias and as such negative attitudes and poor conduct are likely to be underreported. Due to space constraints in the survey, we were unable to account for the effects of different levels or types of training. Finally, while there is preliminary support for the psychometric properties of the novel scales, we were unable to comprehensively evaluate their validity. This would be particularly important with regard to the DFV performance scale as there is no certainty that this self-report measure is reflective of officers’ actual performance.

One potential avenue of investigation would involve examining whether attitudes are primarily seen as individual psychological traits or internal tendencies/feelings. This research could shed light on the underlying mechanisms that shape attitudes within the context of policing. Additionally, exploring the extent to which attitudes are associated with cultural and social norms, as well as organizational structures and processes, would provide valuable insights into how attitudes are influenced by external factors.

Exploring the effects of stress resulting from responding to DFV incidents is another avenue for future investigation. Unraveling the relationships between stress, mental well-being, and organizational performance would help guide effective interventions and support systems.

## Conclusions

The self-report instrument provides an assessment of police officers’ attitudes and stress in relation to DFV incidents, serving as a valuable tool for monitoring changes and evaluating intervention effectiveness over time. It enables us to gain insights, identify areas for improvement, and implement targeted interventions to address the issue effectively. The findings suggest that dissatisfaction and suboptimal police responses are likely not caused by gender bias or misunderstanding principally, but instead by the larger political climate that puts pressure on frontline police officers to “solve” DFV and wear many hats. This leads to stress-inducing internal processes and policies, which in turn negatively impact some officers’ attitudes and ability to respond effectively. However, it was noted that the majority of officers viewed DFV as a serious issue and endorsed police intervention. To better serve victims of DFV, it is crucial for police officers to receive support in both performing their duties and managing the challenges of this difficult task.

**Table 6.** Hierarchical Regressions Predicting DFV Stress, Attitudes, and Performance.

Regression variables		Variable statistics		Model statistics			
Dependent variables	Final model	*Β*	*p-*level	Model *R^2^*	Adjusted *R^2^*	Model *F* change	Model *p*-level
DFV stress	** **			.725	.709	19.403**	<.001
	General police stress	0.643	<.001				
	Male gender	−0.042	.409				
	Emotional stability	−0.004	.938				
	Lived experience	−0.124	.012				
	Severity of DFV	0.141	.012				
	Frequency of DFV	0.147	.008				
	DFV attitudes	0.241	<.001				
DFV attitudes				.340	.318	21.771**	<.001
	Male gender	0.132	.061				
	General police stress	−0.008	.943				
	Length of service	−0.211	.002				
	Severity of DFV	−0.3	<.001				
	DFV stress	0.52	<.001				
DFV performance				.290	.238	12.702**	<.001
	General police stress	0.106	.457				
	Male gender	−0.023	.782				
	Length of service	−0.225	.015				
	Rural location	−0.133	.104				
	Emotional stability	0.164	.059				
	Social support	0.171	.049				
	Frequency of DFV	0.120	.214				
	DFV stress	−0.113	.434				
	DFV attitudes	−0.331	.001				

*Note.* DFV = domestic and family violence.

*p < .05. **p < .01.

## References

[bibr1-10778012241239944] Anshel M. H. (2000). A conceptual model and implications for coping with stressful events in police work. Criminal Justice and Behavior, 27(3), 375–400. 10.1177/0093854800027003006

[bibr2-10778012241239944] Balenovich J. Grossi E. Hughes T. (2008). Toward a balanced approach: Defining police roles in responding to domestic violence. American Journal of Criminal Justice, 33(1), 19–31. 10.1007/s12103-007-9028-5

[bibr3-10778012241239944] Barrett E. C. Hamilton-Giachritsis C. (2013). The victim as a means to an end: Detective decision making in a simulated investigation of attempted rape. Journal of Investigative Psychology and Offender Profiling, 10(2), 200–218. 10.1002/jip.1385

[bibr4-10778012241239944] Belknap J. (1995). Law enforcement officers’ attitudes about the appropriate responses to woman battering. International Review of Victimology, 4(1), 47–62. 10.1177/026975809500400104

[bibr5-10778012241239944] Bianchi R. (2018). Burnout is more strongly linked to neuroticism than to work-contextualized factors. Psychiatry Research, 270, 901–905. 10.1016/j.psychres.2018.11.015 30551342

[bibr6-10778012241239944] Chan J. (2001). Negotiating the field: New observations on the making of police officers. Australian & New Zealand Journal of Criminology, 34(2), 114–133. 10.1177/000486580103400202

[bibr7-10778012241239944] Cohen S. Mermelstein R. Kamarck T. Hoberman H. M. (1985). Measuring the functional components of social support. In Sarason I. G. Sarason B. R. (Eds.), Social support: Theory, research and applications (pp. 73–94). Springer.

[bibr8-10778012241239944] DeJong C. Burgess-Proctor A. Elis L. (2008). Police officer perceptions of intimate partner violence: An analysis of observational data. Violence and Victims, 23(6), 683–696. 10.1891/0886-6708.23.6.683 19069561

[bibr9-10778012241239944] Dollard M. F. McTernan W. (2011). Psychosocial safety climate: A multilevel theory of work stress in the health and community service sector. Epidemiology and Psychiatric Sciences, 20(4), 287–293. 10.1017/S2045796011000588 22201204

[bibr10-10778012241239944] El Sayed S. A. DeShay R. A. Davis J. B. Knox K. N. Kerley K. R. (2022). A blue step forward: An exploratory study of law enforcement perceptions of intimate partner violence in the southern United States. Journal of Interpersonal Violence, 37(9-10), Article 6534. 10.1177/0886260520966675 33084493

[bibr11-10778012241239944] Gill C. Campbell M. Ballucci D. (2019). Police officers’ definitions and understandings of intimate partner violence in New Bruswick, Canada. Police Journal: Theory, Practice and Principles, 94(1). 10.1177/0032258X19876974

[bibr12-10778012241239944] Goodman-Delahunty J. Crehan A. C. (2016). Enhancing police responses to domestic violence incidents: Reports from client advocates in New South Wales. Violence Against Women, 22(8), 1007–1026. 10.1177/1077801215613854 26567295

[bibr13-10778012241239944] Gosling S. D. Rentfrow P. J. Swann W. B. Jr. (2003). A very brief measure of the big five personality domains. Journal of Research in Personality, 37, 504–528. 10.1016/S0092-6566(03)00046-1

[bibr14-10778012241239944] Gracia E. Martín-Fernández M. Marco M. Santirso F. A. Vargas V. Lila M. (2018). The willingness to intervene in cases of intimate partner violence against women (WI-IPVAW) scale: Development and validation of the long and short versions. Frontiers in Psychology, 9, 1146. 10.3389/fpsyg.2018.01146 30065678 PMC6056762

[bibr15-10778012241239944] Hardy M. E. Conway M. E. (1988). Role theory: Perspectives for health professionals (2nd ed.). Appleton & Lange.

[bibr16-10778012241239944] Hegarty K. Hindmarsh E. D. Gilles M. T. (2000). Domestic violence in Australia: Definition, prevalence and nature of presentation in clinical practice. The Medical Journal of Australia, 173(7), 363–367. 10.5694/j.1326-5377.2000.tb125688.x 11062792

[bibr17-10778012241239944] Huey L. Ricciardelli R. (2015). This isn’t what I signed up for’: When police officer role expectations conflict with the realities of general duty police work in remote communities. International Journal of Police Science & Management, 17(3), 194–203. 10.1177/1461355715603590

[bibr18-10778012241239944] Islam J. Mazerolle P. (2022). Nexus between police attitudes and responses to domestic and family violence in Australia: Does training matter? Policing and Society, 32(10), 1–16. 10.1080/10439463.2022.2029436

[bibr19-10778012241239944] Johnson R. R. (2004). Police officer frustrations about handling domestic violence calls. The Police Journal: Theory, Practice and Principles, 77(3), 207–219. 10.1350/pojo.77.3.207.54090

[bibr20-10778012241239944] Kebbell M. R. (2022). Police are influenced by anchoring and risk when allocating resources for scenario-based intimate partner violence cases. Journal of Interpersonal Violence, 37(17-18), NP16377–NP16396. 10.1177/08862605211021974 34098801

[bibr21-10778012241239944] Kop N. Euwema M. Schaufeli W. (1999). Burnout, job stress and violent behaviour among Dutch police officers. Work & Stress, 13(4), 326–340. 10.1080/02678379950019789

[bibr22-10778012241239944] Loftus B. (2009). Police culture in a changing world. Oxford University Press.

[bibr23-10778012241239944] Maple E. Kebbell M. (2021). Responding to domestic and family violence: A qualitative study on the changing perceptions of frontline police officers. Violence Against Women, 27(12-13), 2377–2398. 10.1177/1077801220975483 33357013

[bibr24-10778012241239944] McCreary D. R. Thompson M. M. (2006). Development of two reliable and valid measures of stressors in policing: The operational and organizational police stress questionnaires. International Journal of Stress Management, 13(4), 494–518. 10.1037/1072-5245.13.4.494

[bibr25-10778012241239944] McPhedran S. Gover A. R. Mazerolle P. (2017). A cross-national comparison of police attitudes about domestic violence: A focus on gender. Policing: An International Journal of Police Strategies & Management, 40(2), 214–227. 10.1108/PIJPSM-06-2016-0083

[bibr26-10778012241239944] Meltzer L. S. Huckabay L. M. (2004). Critical care nurses’ perceptions of futile care and its effect on burnout. American Journal of Critical Care, 13(3), 202–208. 10.4037/ajcc2004.13.3.202 15149054

[bibr27-10778012241239944] Meyer S. (2011). Seeking help for intimate partner violence: Victims’ experiences when approaching the criminal justice system for IPV-related support and protection in an Australian jurisdiction. Feminist Criminology, 6(4), 268–290. 10.1177/0004865812443677

[bibr28-10778012241239944] Myhill A. Johnson K. (2016). Police use of discretion in response to domestic violence. Criminology & Criminal Justice, 16(1), 3–20. 10.1177/1748895815590202

[bibr29-10778012241239944] Ozbay F. Johnson D. C. Dimoulas E. Morgan C. A. Charney D. Southwick S. (2007). Social support and resilience to stress: From neurobiology to clinical practice. Psychiatry (Edgmont), 4(5), 35–40. PMCID: PMC2921311. PMC2921311 20806028

[bibr30-10778012241239944] Papazoglou K. Blumberg D. M. Chiongbian V. B. Tuttle B. M. Q. Kamkar K. Chopko B. Milliard B. (2020). The role of moral injury in PTSD among law enforcement officers: A brief report. Frontiers in Psychology, 11(310). 10.3389/fpsyg.2020.00310 PMC7064734 32194477

[bibr31-10778012241239944] Pereira Vieira C. Coelho R. Manuel Costa P. Nunes C. (2022). The influence of law enforcement officers’ sex in their attitude toward intimate partner violence situations. Women & Criminal Justice. 10.1080/08974454.2022.2143739

[bibr32-10778012241239944] Pole N. (2008). Predictors of PTSD symptoms in police officers: From childhood to retirement. In Delahanty D. L. (Ed.), The psychobiology of trauma and resilience across the lifespan (pp. 47–67). Jason Aronson.

[bibr33-10778012241239944] Resnick S. Rosenheck R. (2008). Integrating peer-provided services: A quasi-experimental study of recovery orientation, confidence, and empowerment. Psychiatric Services, 59(11), 1307–1314. 10.1176/ps.2008.59.11.1307 18971407

[bibr34-10778012241239944] Robinson A. L. Chandek M. S. (2000). The domestic violence arrest decision: Examining demographic, attitudinal, and situational variables. Crime & Delinquency, 46(1), 18–37. 10.1177/0011128700046001002

[bibr35-10778012241239944] Segrave M. Wilson D. Fitz-Gibbon K. (2018). Policing intimate partner violence in Victoria (Australia): Examining police attitudes and the potential of specialisation. Australian & New Zealand Journal of Criminology, 51(1), 99–116. 10.1177/0004865816679686

[bibr36-10778012241239944] Serrano-Montilla C. Garrido-Macías M. Sáez-Díaz J. (2023). Assessing police attitudes toward intervention in gender violence: The role of training, perceived severity, and myths about intimate partner violence against women. Journal of Family Violence. 10.1007/s10896-023-00605-8

[bibr37-10778012241239944] Sinden P. G. Stephens J. B. (1999). Police perceptions of domestic violence: The nexus of victim, perpetrator, event, self and law. Policing: An International Journal of Police Strategies & Management, 22(3), 313–327. 10.1108/13639519910285071

[bibr38-10778012241239944] Stalans L. Finn M. (2000). Gender differences in officers’ perceptions and decisions about domestic violence cases. Women & Criminal Justice, 11(3), 1–24. 10.1300/J012v11n03_01

[bibr39-10778012241239944] Stanetić K. Tesanović G. (2013). Influence of age and length of service on the level of stress and burnout syndrome. Medicinski Pregled, 66(3-4), 153–162. 10.2298/mpns1304153s 23653994

[bibr40-10778012241239944] Stark E. (2007). *Coercive control: The entrapment of women in personal life*. https://ebookcentral-proquest-com.libraryproxy.griffith.edu.au

[bibr41-10778012241239944] Stark E. (2012). Looking beyond domestic violence: Policing coercive control. Journal of Police Crisis Negotiations, 12(2), 199–217. 10.1080/15332586.2012.725016

[bibr42-10778012241239944] Stewart C. Langan D. Hannem S. (2013). Victim experiences and perspectives on police responses to verbal violence in domestic settings. Feminist Criminology, 8(4), 269–294. 10.1177/1557085113490782

[bibr43-10778012241239944] Sun I. (2007). Policing domestic violence: Does officer gender matter? Journal of Criminal Justice, 35, 581–595. 10.1016/j.jcrimjus.2007.09.004. 10.1016/j.jcrimjus.2007.09.004

[bibr44-10778012241239944] Zhao R. Zhang H. Jiang Y. Yao X. (2018). The tendency to make arrests in domestic violence: Perceptions from police officers in China. International Journal of Offender Therapy and Comparative Criminology, 62(16), 4923–4941. 10.1177/0306624(1880165 30253673

